# The Statistical-Mechanical Meaning of the Wave Function of Quantum Mechanics

**DOI:** 10.3390/e28060710

**Published:** 2026-06-20

**Authors:** Alberto Robledo

**Affiliations:** Instituto de Física, Universidad Nacional Autónoma de México, P.O. Box 20-364, Mexico City 01000, Mexico; robledo@fisica.unam.mx

**Keywords:** quantum mechanics, wave functions, statistical mechanics, inhomogeneous systems, classical density functional theory, density fluctuations

## Abstract

We address the paradoxical transformation of a classical-mechanical particle motion when the space and time scales of observation pass below the uncertainty principle threshold. This is analyzed in the language of classical statistical mechanics, considering specifically many-particle systems inhomogeneous along one spatial direction. We employ the density functional formalism in its square-gradient form and find: (i) The macroscopic solution is analogous to the classical trajectory of a particle under a potential of force given by (minus) the free energy density. Whereas, (ii) fluctuations around the solution in (i) are equal to the quantum-mechanical wave functions of a particle under a potential given by the curvature of the free energy density. We illustrate this situation with three textbook examples: A particle in a box, the harmonic oscillator, and the hydrogen atom. We show that their time-independent Schrödinger equation wave functions describe, respectively, the fluctuations of a fluid interface, of critical point fluctuations, and of a confined ideal gas. At large scales, sharp probability distributions make fluctuations irrelevant; the vanishing of the first variation yields the macroscopically observable statistical-mechanical non-uniformity, equivalent to the classical particle trajectory. But at sufficiently small scales, with necessarily very few particles, distributions appear much wider, fluctuations dominate, and one obtains the Schrödinger equation (for the microscopic potential).

## 1. Introduction

As many others over many years, we consider the limit of validity of classical mechanics when the space and time scales of observation are amplified beyond the point established by the uncertainty principle [1]. Our aim is to establish a theoretical footing on which to gain a true understanding of the appearance of quantum mechanical features that manifest below this boundary. We analyze this general phenomenon in the language of *classical* statistical mechanics via a strict analogy that holds between these two branches of physics. Connections between statistical mechanics and quantum mechanics have been known and explored extensively for a long time; see, for example, Ref. [2] and references therein. But here we address a different and unused version with consequential features that offer a new physically satisfactory understanding. These new properties and attributes originate from the appearance of two different mechanical analogs, one for the classical particle and another for the quantum particle. These analogs are of dissimilar nature since their potential functions differ; that for the former corresponds to (minus) the statistical-mechanical free energy density, while that for the latter is the curvature of the same statistical-mechanical quantity. As we shall see, the perplexing contrast between classical and quantum particle mechanics corresponds, in statistical-mechanical language, to the distinction between the macroscopic thermodynamic potential and the microscopic interactions between the constituent degrees-of-freedom.

The formal resemblance between statistical and quantum mechanics has been known, studied and applied already over many decades, in particular, in the form between statistical mechanics (SM) of fields and quantum field theory (QFT), see [3] and references therein. We shall not give a detailed account of the developments based on this recognition, which up to this date has produced a very large number of published studies [4]. Instead, we present briefly general informative comments to help demarcate our particular advance that, as we shall see later, differs in important mathematical and physical issues from previous studies. The mathematical expressions that portray most clearly the resemblance between SM and QFT are those that represent the partition function for the former (with the sum over configurations generalized as integrals over continuum variables) and the path integral formulation of QFT. These expressions point out the similar roles played by the free energy in SM of fields and the action in QFT [3]. Most contributions from QFT to SM have focused on the scaling properties of critical phenomena via the concept and the method of renormalization. On the other hand, SM has provided QFT with general interpretations in terms of elementary particles and quasi-particles. Mathematically, we can distinguish two main connections between QFT and SM: the first, referred to as the Minkowski QFT, relates a d−1 spatial and one time dimension system with a SM system described by a quantum Hamiltonian [3]. The second, the Euclidean QFT, relates a *d*-dimensional space system with an SM classical system [3]. As a difference, here we follow a standard optimization procedure in the SM of inhomogeneous systems, within classical free energy density functional theory [5,6], that involves, as a first step, the vanishing of the first variation (the associated Euler–Lagrange equation) and as a second step the determination of the second variation to establish the stability of the stationary solution obtained in the first step. These two steps provide, separately, the connections with classical and quantum mechanics.

Specifically, we consider statistical-mechanical systems composed of many degrees of freedom (say, particles) for which the equilibrium (more generally, stationary) states are inhomogeneous along only one spatial direction *r*. We determine these states through the free energy density functional formalism in its most accessible form, via the phenomenological square-gradient approach [5,6]. We recall [5,6] that f[ρ(r)], the Helmholtz free energy density term in the square-gradient density functional expression, is written down by replacing the constant density $\rho_{u}$ in the uniform stationary free energy solution by a generic non-uniform density profile ρ(r). As known in [7,8,9], the statistical-mechanical macroscopic solution is given by the classical mechanical trajectory of a single particle when its position *R* at time τ is translated into the statistical-mechanical system’s density ρ at the spatial position *r*. The potential of force U(R) acting on the classical particle is given by (minus) the grand potential density ω(ρ)=f(ρ)−μρ, where μ is the chemical potential. On the other hand, the fluctuations about the non-uniform stationary solution $\rho_{s}\left(r\right)$ are found [7,8,9] to be equal to the wave functions ψ(r) of the time-independent Schrödinger equation for a single particle under a potential V(r) given by the curvature of the free energy f[ρ(r)], $V\left(r\right)=\partial^{2}f\left[\rho \left(r\right)\right]/\partial \rho {\left(r\right)}^{2}$ [7,8,9]. It is important to point out [9] that the said inhomogeneous system fluctuations, and likewise the wave functions of the equivalent single-particle quantum problem, involve not only the direction of inhomogeneity *r*, but also the remaining space coordinates. So, for example, an inhomogeneity in three-dimensional space in Cartesian r=(x,y,z), cylindrical r=(r,ϕ,z), spherical r=(r,θ,ϕ), or other, spatial coordinates, lead to fluctuations, or wave functions ψ(r) dependent on all three coordinates [9]. We illustrate this situation with three textbook quantum-mechanical examples: A particle in a box, the harmonic oscillator, and the hydrogen atom. We show that their wave functions describe, respectively, the fluctuations of a macroscopic fluid interface, those of a critical point fluctuation, and those of a confined ideal gas.

We discuss our results but advance upfront the following equilibrium statistical-mechanical comment: As always observed, at large scales of space and time, the presence of many degrees of freedom makes fluctuations irrelevant, as in these conditions they are minuscule and ephemeral and probability distributions extremely sharp. The vanishing of the first variation yields the observable statistical-mechanical non-uniformity, which translates, as mentioned, in mechanics language into the classical particle trajectory. On the contrary, at sufficiently small space and time scales, the probability distributions have a much wider appearance and fluctuations dominate when only a few degrees of freedom are present. One obtains the time-independent Schrödinger equation but for a *different*, but compatible, microscopic potential, a potential acting on a single degree of freedom. In relation to the above, the Principle of Least Action (PLA) [10] has historically established hidden connections amongst problems that belong to different branches of physics. As a first step, we make use of it to determine stationary spatially-inhomogeneous statistical-mechanical systems, together with their classical-mechanical analogues. The second step, the stability analysis of the inhomogeneous stationary states obtained from the Euler–Lagrange equation [11], requires the determination of the infinite variations of density fluctuations, considered, for instance, in path integral methods. Or, as we emphasize here, density fluctuations are structured, organized, and physically interpreted by the wave functions of the time-independent Schrödinger equation [12]. This time we encounter quantum-mechanical analogs, but with a different potential function. This circumstance provides access to advance physical interpretations.

Before the closing remarks, in the previous to last Section 6, we depart from our one-particle time-independent Schrödinger equation description. There we speculate on how the perspective presented could possibly be generalized in the future to be applicable to and provide viewpoints on topics such as measurement collapse, entanglement, wave function dynamics, non-locality, and others.

## 2. A Phenomenological Density Functional and Its Euler–Lagrange Equation

A widely employed (grand canonical) free energy density functional to study inhomogeneous statistical-mechanical systems [5,6], written in terms of fluid applications, is

(1) $$\Omega \left[\rho \left(r\right)\right]=\int \{f\left[\rho \left(r\right)\right]-\mu \rho \left(r\right)+A|\nabla \rho (r){|}^{2}dr\},$$

where f[ρ(r)] is the Helmholtz free-energy expression per unit volume of a uniform fluid of density $\rho_{u}$ with that density replaced by an arbitrary density function ρ(r), where r is the spatial position. In Equation (1) μ is the chemical potential, and *A* is related to the direct correlation function of a uniform fluid again with the same density replacement [5,6], here assumed to be a constant. The derivation of Equation (1) is of phenomenological nature, whereas many other density functional expressions have been derived from first principles. See, for example, Refs. [13,14,15,16]. The vanishing of the first variation of Equation (1), its associated Euler–Lagrange equation, is [9]

(2) $$\frac{\delta \Omega }{\delta \rho (r)}=\frac{\partial f}{\partial \rho }-\mu -2A\nabla^{2}\rho (r)=0.$$

The possible solutions of this equation are denoted as $\rho_{s}\left(r\right)$, and called stationary solutions.

When we consider a system that is inhomogeneous only in one direction *r* Equation (2) becomes [9]

(3) $$\frac{d^{2}\rho }{dr^{2}}=-\frac{dU}{d\rho },$$

where U(ρ)=−f(ρ)+μρ, and the factor 2A has been taken to be unity. This can be seen as corresponding to Newton’s second law of motion for a classical particle of unit mass. In this mathematical analogy, the density ρ plays the role of the position *R* of the particle; the spatial coordinate *r* along the inhomogeneity plays the role of time τ and U(ρ) is the potential of force that governs the motion of the particle. Thus, a uniform bulk state, where the density is constant (dρ/dr=0), corresponds to a classical particle at rest (or moving with constant velocity when observed from a different frame of reference) at a minimum (or maximum) of the potential U(ρ). Likewise, the spatial variation in the density profile across an interface, such as a liquid-gas interface, translates into the trajectory ρ(r) of this particle as it moves between potential hills and valleys.

**Figure 1:**
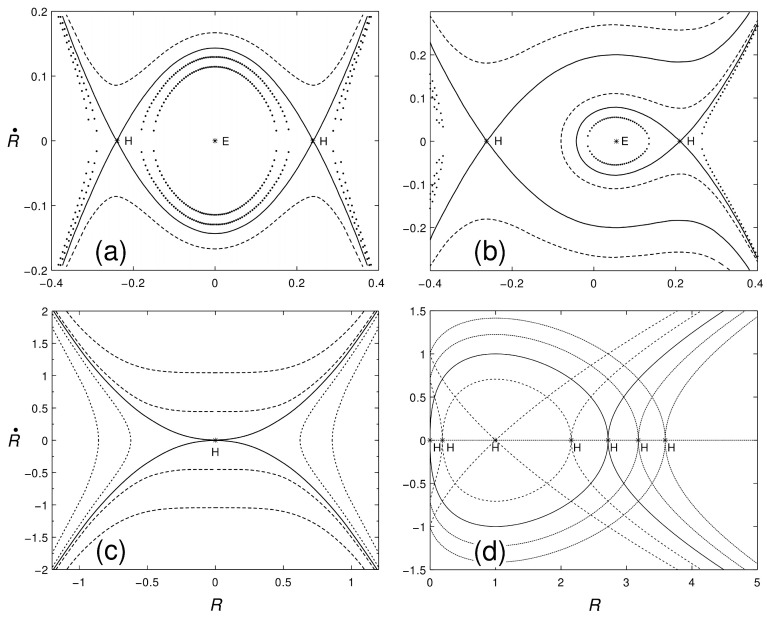
Phase portraits (Equation (4)) for the specific model examples described in the text. *R* and $\dot{R}$ denote particle position and velocity, respectively, equivalent to density ρ and its spatial derivative dρ/dr, respectively. Solid lines correspond to zero total energy particle trajectories equivalent to macroscopic inhomogeneities in one direction. Labels *H* and *E* denote hyperbolic and elliptic points, respectively. A solid line that contains one or more *H* points is named a separatrix. (**a**) A separatrix segment joining two *H* points corresponds to a two-phase coexistence interface. (**b**) A loop around an *H* point represents a critical radius cylindrical column or a critical radius spherical nucleation droplet. (**c**) Curves passing through the *H* point describe a macroscopic dominant critical point fluctuation. (**d**) Level curve segments that correspond to negative free energy curvature describe a confined ideal gas within a spherical cavity or spherical shell. See text for details.

A first integration of Equation (3) yields the phase portrait (momentum-position relationship) associated with the classical particle ρ→R, r→τ,

(4) $$\dot{R}\equiv \frac{d\rho }{dr}=\pm \sqrt{U_{0}-U\left(R\right)},$$

where $U_{0}$ is an integration constant representing the total energy of the particle. In Figure 1 we show the phase portraits for the classical-mechanical particle analogs of the three examples detailed below in Section 4. Their potentials of force U(R) are as follows: (i) Figure 1a: A parabolic well between two parabolic hills of equal height. First parabolic hill, $U\left(R\right)=-a{\left(R+R_{1}\right)}^{2},R<-R_{0}$; parabolic well, $U\left(R\right)=a_{1}R^{2}-a_{0},-R_{0}<R<R_{0}$; second parabolic hill, $U\left(R\right)=-a{\left(R-R_{1}\right)}^{2},R_{0}<R$, $a_{0}=aR_{1}\left(R_{0}-R_{1}\right),a_{1}=a\left(R_{0}-R_{1}\right)/R_{0}$. See [9]. (ii) Figure 1b: Same as Figure 1a, except that the parabolic hills have different heights. (iii) Figure 1c: A quartic (zero curvature) well, $U\left(R\right)=-bR^{4}$. (iv) Figure 1d: A well of the form U(R)=−RlnR+(1+μ)R.

## 3. Density Fluctuations and the Time-Independent Schrödinger’s Equation

In optimization problems, the most common procedure is to calculate the first derivative of a functional and set it to zero to find an extremum. Since this often yields a physically sensible solution, it is common to stop at this step. However, a complete physical understanding requires the stability of the solutions of the Euler–Lagrange equation to be determined. That is, clarifying whether each solution constitutes a minimum, a maximum, or a saddle point in the functional space under consideration, in our case, the space of inhomogeneous densities ρ(r). This demands an examination of the second variation in the functional [17].

To analyze stability, we consider fluctuations around the stationary solution $\rho_{s}\left(r\right)$ obtained from the Euler–Lagrange Equation (2). We write an unspecified density as

(5) $$\rho \left(r\right)\equiv \rho_{s}\left(r\right)+\delta \rho \left(r\right),$$

where δρ(r) represents an arbitrary deviation from the stationary state density. The second variation of the grand potential Ω is given by [9]:

(6) $$\delta^{2}\Omega =\frac{1}{2}\int \delta \rho \left(r\right)\left[\frac{\partial^{2}f}{\partial \rho^{2}}{|}_{\rho_{s}\left(r\right)}\delta \rho \left(r\right)-2A\nabla^{2}\delta \rho \left(r\right)\right]dr.$$

If the stationary solution $\rho_{s}\left(r\right)$ is a stable minimum, any fluctuation δρ should increase the free energy, $\delta^{2}\Omega >0$. But if there is a fluctuation, or a specific set of them, for which $\delta^{2}\Omega <0$ a process will be triggered, taking the inhomogeneous system away from $\rho_{s}$. Such fluctuations lead to an important understanding of the nature of the statistical-mechanical system under consideration. With this objective in mind, we decompose the arbitrary fluctuation δρ(r) into a complete basis set of functions $\left\{\psi_{n}\left(r\right)\right\}$:

(7) $$\delta \rho \left(r\right)=\sum_{n}C_{n}\psi_{n}\left(r\right).$$

Substituting the decomposed fluctuation into the expression for $\delta^{2}\Omega$ and requiring the result to be stationary with respect to the coefficients $C_{n}$ leads to an eigenvalue equation for the basis functions $\psi_{n}\left(r\right)$ [9]:

(8) $$-A\nabla^{2}\psi_{n}\left(r\right)+\frac{1}{2}\frac{\partial^{2}f}{\partial \rho^{2}}{|}_{\rho_{s}\left(r\right)}\psi_{n}\left(r\right)=E_{n}\psi_{n}\left(r\right).$$

Equation (8) is formally identical to the one-particle time-independent Schrödinger equation. In this analogy, the term $-A\nabla^{2}$ corresponds to the kinetic energy operator $\left(-\hbar^{2}/2m\right)\nabla^{2}$; the term $\frac{1}{2}\left(\partial^{2}f/\partial \rho^{2}\right){|}_{\rho_{s}}$ plays the role of the external potential V(r) and the eigenvalues $E_{n}$ are the associated energy levels.

With this identification, the second variation of the free energy can be rewritten as follows:

(9) $$\delta^{2}\Omega =\sum_{n}E_{n}{\left(C_{n}\psi_{n}\left(r\right)\right)}^{2},$$

and since all the ${\left(C_{n}\psi_{n}\left(r\right)\right)}^{2}>0$, the stability of the stationary statistical-mechanical inhomogeneous state $\rho_{s}\left(r\right)$ is entirely determined by the eigenvalues $E_{n}$. If all $E_{n}>0$, $\delta^{2}\Omega >0$ and the solution is a stable minimum. A negative eigenvalue signals an instability, and its associated eigenfunction $\psi_{n}\left(r\right)$ carries information about the nature of that instability. A vanishing eigenvalue indicates an interesting singular property contained in its associated eigenfunction.

Thus, the analysis of fluctuations in all classical, inhomogeneous (in one coordinate direction), statistical-mechanical systems, described in terms of Equation (1), encompasses the entire structure of time-independent, single-particle, quantum mechanics. The eigenfunctions $\psi_{n}\left(r\right)$ of the fluctuation modes are mathematically equivalent to the stationary quantum mechanical wave eigenfunctions, and their associated eigenvalues $E_{n}$ correspond to the quantized energies of the system. As indicated by Equation (7), all fluctuations δρ(r) of the inhomogeneous system can be obtained as superpositions of the eigenfunctions $\psi_{n}\left(r\right)$. Notice that the quantum-mechanical potential function V(r) in the Schrödinger Equation (8) is given by the second derivative of the free energy density, $\frac{1}{2}\left(\partial^{2}f/\partial \rho^{2}\right){|}_{\rho_{S}}$, whereas the classical-mechanical potential of force in the previous section is given by (minus) the free energy density itself, −f(ρ)+μρ.

*Time Evolution of Inhomogeneous System Fluctuations.* Having established the stability of the stationary states of inhomogeneous systems, we now turn to the time evolution of their fluctuations, expressed as $\delta \rho \left(r,t\right)=\rho \left(r,t\right)-\rho_{s}\left(r\right)$. A standard approach, following Landau’s formalism for slow relaxation of small fluctuations, is to describe this evolution by a *dissipative* kinetic equation [18]. A typical form for this equation, compatible with the Euler–Lagrange Equation (2) we have employed, is [9]:

(10) $$\frac{\partial \delta \rho (r,t)}{\partial t}=-M\left(\frac{\partial^{2}f}{\partial \rho^{2}}{|}_{\rho_{s}\left(r\right)}-2A\nabla^{2}\right)\delta \rho \left(r,t\right),$$

where *M* is a mobility coefficient and *f* is the Helmholtz free energy density.

To solve this equation, we expand, as before, the fluctuation δρ(r,t) in the complete basis of eigenfunctions $\psi_{n}\left(r\right)$

(11) $$\delta \rho \left(r,t\right)=\sum_{n}C_{n}\left(t\right)\psi_{n}\left(r\right).$$

Substituting this expansion into the kinetic equation yields a simple decoupled equation for each mode amplitude

(12) $$\frac{dC_{n}\left(t\right)}{dt}=-ME_{n}C_{n}\left(t\right),$$

where $E_{n}$ is the eigenvalue associated with the eigenfunction $\psi_{n}\left(r\right)$. This is a straightforward differential equation with an exponential solution $C_{n}\left(t\right)\propto e^{-ME_{n}t}$, where the sign of the eigenvalue $E_{n}$ determines whether the fluctuation decays (stable mode) or grows (unstable mode) over time.

Notice that the use of Equation (10) imposes non-unitary wave function time evolution. In Section 6 we comment on this choice and indicate alternatives, including probability conservation along time evolution.

## 4. Three Examples

### 4.1. Particle in a Well and a Liquid-Gas Interface

Our first illustrative example is a particle in a well that, as we shall see, corresponds, macroscopically, to a model interfacial density profile for liquid-gas phase coexistence, or, more generally, a metastable two-phase interface. To this end, we chose a piecewise parabolic continuous model for the grand potential free energy density ω(ρ)=f(ρ)−μρ in Equation (1) [9] representative of a fluid system at a temperature and chemical potential for which there is two-phase coexistence. The homogeneous (mean-field) grand potential density ω(ρ) exhibits two equal-height minima separated by a maximum. Variations of temperature and/or chemical potential modify the minima heights and introduce metastability. The model consists of three continuously joined parabolas: one centered at the unstable maximum and one for each of the two stable minima. The planar equilibrium density profile ρ(x) is then found by solving the Euler–Lagrange Equation (3) in Cartesian coordinates (x,y,z). See Figure 1 in [9]. The piecewise parabolic free-energy density model involves a modification of the fluid phase-coexistence grand potential free energy density ω(ρ) through appropriate changes of variables such that the two equal-height minima occur at new ‘densities’ $\phi_{1}$ and $\phi_{2}=-\phi_{1}$ and the intermediate maximum at the midpoint $\phi_{0}=0$. See Ref. [9] for details. This transformation is similar to that relating the lattice gas grand potential free energy density ω(ρ) to the Ising ferromagnet free energy f(m), both under the mean field approximation, and where the magnetization *m* for subcritical temperatures and vanishing external field exhibits two minima at $m_{1}$ and $m_{2}=-m_{1}$ and a maximum at $m_{0}=0$.

The classical-mechanical particle trajectory analogue to the macroscopic interface can be visualized by considering that, in this example, the potential of force U(ρ)=−ω(ρ)=−f(ρ)+μρ consists of two equal-height hills (maxima) separated by a valley (minimum). Recalling that the particle’s position *x* at time *t* is given by replacing the fluid density ρ at location *x*, that is ρ⇒x and x⇒t, the trajectory begins to run down infinitely slowly from the (gas bulk phase) maximum, acquires speed as it falls, traverses the potential well, and climbs up with diminishing velocity towards the second maximum until it reaches, again infinitely slowly, the liquid bulk phase. This trajectory corresponds to the vanishing total (kinetic plus potential) energy represented by the separatrix ($U_{0}=0$) in Figure 1a. Other trajectories can be taken out from Figure 1a,b and subsequently their statistical-mechanical analogs established.

Now we turn to look at the statistical-mechanical stability of the interfacial density profile obtained from the Euler–Lagrange equation when particularized to the piecewise parabolic continuous model. The stability analysis transforms immediately into the well-known problem of a quantum particle in a finite-height potential well. The potential function V(r) in the Schrödinger Equation (8) consists of the tree curvature values of the parabolas, one negative $V_{0}$ finite space enclosed within a positive $V_{1}=V_{2}$ extended region. This has been determined first in Cartesian r=(x,y,z), then in cylindrical r=(r,ϕ,z), and finally in spherical r=(r,θ,ϕ) coordinates [9]. For each case, it yields different physical insights into interfacial phenomena [9].

When solved in Cartesian coordinates, equilibrium phase coexistence, the separatrix in Figure 1a, the first eigenvalue of Equation (8) is equal to zero, and all others are positive [9]. The corresponding first eigenfunction represents a rigid displacement of the planar interface (the Euler–Lagrange solution of Equation (3)), while the rest can be identified with other types of interfacial distortions. This means that the most relevant fluctuations of the planar interface are (free energy) costless rigid position shifts. The interfacial density profile is a soliton—a stable, non-dispersive wave—that, when the temperature or the chemical potential are tuned to favor one phase over the other, the soliton propagates, representing the condensation or evaporation of one phase at the expense of the other [14].

Solving the problem in cylindrical coordinates, macroscopically a cylindrical column of, say, liquid surrounded by gas, one finds that the first eigenvalue is negative, but all others are positive. The eigenfunction related to the negative eigenvalue is an undulation of the cylindrical interface with a wavelength equal to the cylinder cross section [9]. The existence of this fluctuation mode is the precursor of the Plateau-Rayleigh instability, the mechanism responsible for the breakup of a cylindrical liquid jet into droplets [19].

Finally, when the problem is formulated in spherical coordinates, it describes the stability analysis of a spherical droplet of liquid surrounded by a metastable gas, or vice versa. There is only one negative eigenvalue, and its eigenfunction corresponds to the interfacial fluctuation mode that changes the droplet radius [9]. The radius being the critical value that corresponds to the Young–Laplace equation [20] in classical nucleation theory [21]. If the droplet’s radius is smaller than this critical value, the fluctuation leads to its collapse; if it is larger, the fluctuation leads to unbounded growth and a full phase transition. All other eigenfunctions, associated with positive eigenvalues, represent stable fluctuation modes that distort the interfacial region in different ways, without triggering droplet collapse or phase change [9].

**Figure 2:**
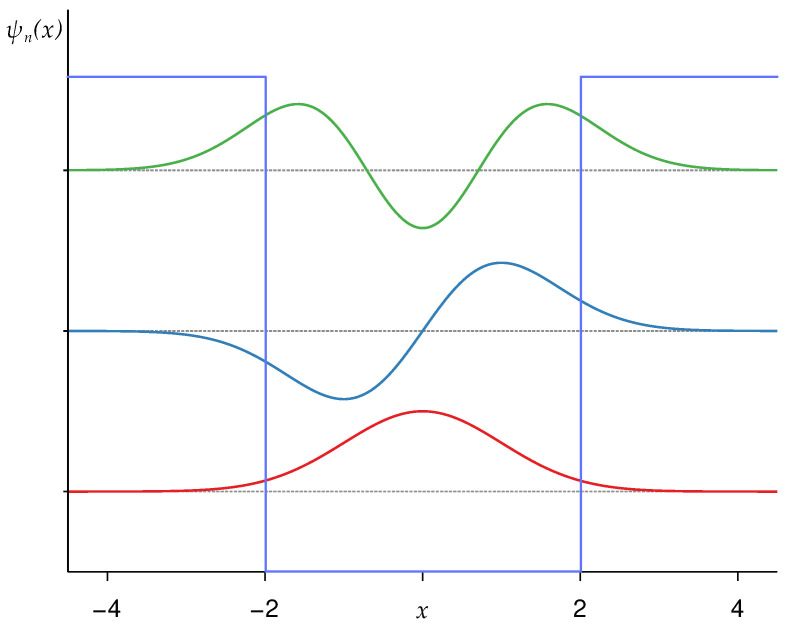
First three eigenfunctions of a particle in a finite-height well potential. The eigenfunctions represent specific types of density fluctuations for a two-phase interface. The first eigenfunction represents a rigid displacement of the interface, the second constitutes an expansion (or shrinkage) of the interface, etc. For a planar interface, the first eigenvalue is zero and signals free interfacial displacements. For a cylindrical interface, the first eigenvalue is negative and the corresponding density fluctuation relates to the Plateau–Rayleigh instability. For a spherical interface, the first eigenvalue is also negative and the associated density fluctuation triggers phase change via nucleation. See text for details.

The finite height well and its firs three eigenfunctions are shown in Figure 2.

Notice that the model interface we have considered displays a well-known mean-field feature, a free energy maximum within a density interval with negative free-energy curvature. For a uniform state, this implies a violation of the second law of thermodynamics, but for a nonuniform state, this is not the case, and actually provides the structure and surface tension of the equilibrium interface between two bulk phases.

The stability analysis of the interfacial problems studied with the piecewise parabolic model led to the determination of the effect of fluctuations on the stationary interfacial density profiles. These fluctuations are identified as the wave functions of a particle on a finite-height well potential, the eigenvalue spectrum of which consists of a number of discrete states below a continuum [14]. The well potential exhibits bound and extended states as well as tunneling. On the other hand, the macroscopic interfacial problem exhibits the Raleigh instability and phase change via nucleation. Our analysis suggests a connection between the macroscopic and microscopic properties described.

### 4.2. The Harmonic Oscillator and Critical Fluctuations

As a second illustrative example, we consider the harmonic oscillator, which, as we show now, appears associated with the description of critical point fluctuations, those that occur in a macroscopic system at a second-order or continuous phase transition. Critical fluctuations display sizes and lifetimes spanning several scales [22]. We denote by ϕ the system order parameter that vanishes ϕ=0 at the critical point of the macroscopic system, that is, in the so-called thermodynamic limit. However, we consider nonuniform fluctuations from ϕ=0 given by stationary (unstable) nonzero functions $\phi_{s}\left(r\right)$, which can be interpreted as local order parameter or magnetization domains, where r is the spatial position.

We initiate our description with a path integral calculation of the partition function *Z* of a large fluctuation at a critical point [23,24] and demonstrate its close connection with our narrative. This is

(13) $$Z=\int_{\Gamma }D\left[\phi \right]\;Z_{\phi },$$

where Γ spans all possible ϕ, while $Z_{\phi }=\exp\left(-F_{c}\left[\phi \right]\right)$ is obtained by summing over the microscopic configurations that lead to a specific form for the order parameter ϕ(r), where its free energy $F_{c}\left[\phi \right]$, or effective action, also named Landau–Ginzburg–Wilson Hamiltonian [25], is

(14) $$F_{c}\left[\phi \right]=a\int_{V}dr\;\left[\frac{1}{2}{\left(\nabla \phi \right)}^{2}+b{\left|\phi \right|}^{\delta_{c}+1}\right].$$

Above, *V* is the volume occupied by the fluctuation, $\delta_{c}$ is the isothermal critical exponent [26], and *a* and *b* are constants. For the largest fluctuation, the path integration over $Z_{\phi }$, which considers all different ϕ(r), is replaced by a saddle-point approximation. This delivers the particular form of ϕ(r) obtained from the dominant configurations [23,24,27]. We refer to this as the dominant fluctuation.

To obtain analytical, closed-form results, we consider the simplest case, a one-dimensional system, r→x, with degrees of freedom, or spins, interacting via long-range forces. This leads to a critical point that complies with the mean-field value $\delta_{c}=3$ [27,28]. Under this conditions the Euler–Lagrange equation obtained from Equation (14) conducts directly to Newton’s second law for a particle in a potential $U\left(\phi \right)=-{b|\phi |}^{\delta_{c}+1}$, where the order parameter ϕ plays the role of position and the spatial coordinate *x* plays the role of time, ϕ→X, x→τ. We obtain

(15) $$\frac{d^{2}\phi }{dx^{2}}=-\frac{dU}{d\phi }.$$

A first integration yields

(16) $$\dot{X}\equiv \frac{d\phi }{dx}=\pm \sqrt{2(C+b|\phi {|}^{\delta_{c}+1})},$$

where *C* is an integration constant representing the total energy of the classical particle.

Varying the values of *C* we obtain the phase portrait in Figure 1c, where each segment of the solid line C=0 starting at the point labeled *H* provides the shape $\phi_{s}\left(x;C=0\right)$ of the largest (macroscopic) dominant fluctuation [27,28]. Notice that the phase portrait in Figure 1c resembles qualitatively the phase portrait of a classical harmonic oscillator (one separatrix crossing at a hyperbolic point *H* the horizontal axis of zero momentum that separates other level curves into four families of nonzero particle total energy, dashed and dotted curves in the figure). But there is an important difference in Figure 1c. The separatrix at *H* has vanishing slope, while that for the classical harmonic oscillator displays cusps at *H* (like the separatrix at the *H* points in Figure 1a,b,d). This difference is crucial; near X=0 the classical particle displacements can grow considerably at almost vanishing velocity, that is, the dominant fluctuation can increase its density considerably at very small density gradients (possibly by filling-up a fractal inner structure [29,30]). On the contrary, the classical harmonic oscillator particle displacements are hampered if velocity is not sharply increased by converting potential into kinetic energy. In the language of the fluid interface, Figure 1a, this translates into a finite interfacial width.

Notice that in this case, there is also a basic difference in thermodynamic terms between the macroscopic uniform system in the thermodynamic limit and the finite-scale nonuniform dominant fluctuation $\phi_{s}\left(x;C\right)$ described. The former displays vanishing order parameter ϕ=0 while the latter has spatial sizes and shapes $\phi_{s}\left(x;C\right)$ given by the phase portrait Equation (16) with different integration constants *C* [27]. The size of the dominant fluctuation diverges when C=0. The nonuniform stationary solutions $\phi_{s}\left(x;C\right)$ can make use, as before, of order parameter intervals with negative free energy curvature.

**Figure 3:**
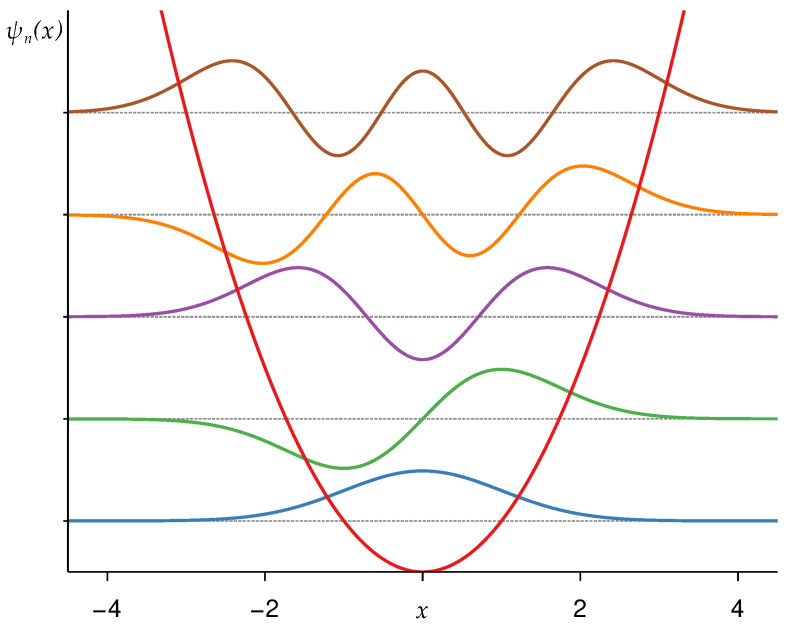
First five eigenfunctions of the harmonic oscillator. The eigenfunctions represent specific types of density perturbations or variations of a dominant critical fluctuation. They are similar to those in Figure 2. for a particle in a well, but important differences are as follows: (a) The entire energy spectrum is discrete, all eigenstates are bounded and nondegenerate, (b) the eigenvalues are evenly spaced, and (c) the first eigenvalue is positive, the zero-point energy. See text for details of the role of these features, in particular, the zero-point energy, in the scale of lifetimes and sizes of critical fluctuations.

As before, the study of the stability of deviations $\delta \phi \left(x\right)\equiv \phi \left(x\right)-\phi_{s}\left(x;C\right)$ off a stationary solution $\phi_{s}\left(x;C\right)$ of Equations (15) and (16), that is, fluctuations about the critical fluctuations, reveals a connection with quantum mechanics, this time with the harmonic oscillator (see Figure 3), since the potential function V(x) in the Schrödinger Equation (8) becomes $V\left(x\right)={b|x|}^{\delta_{c}-1}=b{\left|x\right|}^{2}$. According to the general property of confined quantum-mechanical particles, all eigenvalues are quantized, and in this case, the discrete levels are non-degenerate and equally spaced. The ground state eigenvalue is positive; its value is the zero-point energy. In statistical-mechanical language, a critical fluctuation undergoes constant variations in size and in its average value of the order parameter or magnetization (via local changes of this quantity). The zero-point energy of the oscillator translates into a lowest non-zero free energy per unit area deviation, or lowest bound surface tension deviation (point tension deviation in our d=1 case), that takes place in the dominant fluctuation obtained above from the Euler–Lagrange equation. For equilibrium critical point states that occur at temperatures that provide the system with thermal energy much larger than, say, the thermal energy of molecular vibrations, critical fluctuations can evolve through many time intervals and acquire many sizes. Possibly, this feature indicates the reason for the characteristic abundance of critical fluctuations throughout many spatial and time scales.

### 4.3. The Hydrogen Atom and Confined Gases

Our third illustrative example is the hydrogen atom, which, as we detail, corresponds macroscopically to a simple, inert, dilute gas confined in a cavity. We consider this cavity to be spherical, or more generally, a spherical shell of radii $r_{1}<r_{2}$. The quantum-mechanical hydrogen atom is usually described by resolving the single-particle time-independent Schrödinger Equation (8) in three-dimensional space with the employment of spherical coordinates r=(r,θ,ϕ), together with a potential function V(r) that attracts the particle and depends only and inversely proportionally on the radial coordinate *r*. Here, we ignore the proportionality factor and use V(r)=−1/r. We are interested in determining the macroscopic (statistical-mechanical) inhomogeneous system obtained from the grand potential functional Ω[ρ(r)] in Equation (1) such that its stability analysis results in the well-known hydrogen atom wave functions and eigenvalues.

To this end, we consider the Helmholtz free energy density $f(\rho_{u})$

(17) $$f\left(\rho_{u}\right)=\rho_{u}\ln\left(\rho_{u}\right)-\rho_{u},$$

that can be recognized, ignoring a temperature factor, to be the free energy density of a uniform ideal gas, a representation of a dilute inert gas. If $\rho_{u}$ above is replaced by an arbitrary three-dimensional nonuniform density ρ(r) we obtain the density functional in Equation (1) with f[ρ(r)]=ρ(r)ln(ρ(r))−ρ(r). Considering that the fluid is inhomogeneous only in the direction *r* and proceeding as in Section 2 we obtain Equations (3) and (4) with U(R)=−RlnR+(1+μ)R, where we have again made the replacement ρ→R (together with 2A=1). The corresponding phase portrait is shown in Figure 1d. Technically, in deriving the stationary solutions $\rho_{s}\left(r;U_{0}\right)$ of the confined ideal gas, it may be necessary to use boundary conditions since the gas exerts a force on the wall of the cavity, or, if it is the case, on the walls of a spherical shell cavity. These boundary conditions can be expressed as

(18) $$\frac{d\rho }{dr}{|}_{r_{1,2}}=\pm \left(\mu -\mu_{1,2}\right),$$

where $r_{1,2}$ and $\mu_{1,2}$ are the radii and the (delta-function-like) wall chemical potentials of the spherical shell, respectively, of the walls 1 and 2 [31].

We look now at the stability of the stationary states specific to this example. That is, we consider the fluctuations $\delta \rho \left(r,\theta ,\phi \right)\equiv \rho \left(r,\theta ,\phi \right)-\rho_{s}\left(r;U_{0}\right)$ of the stationary solutions of the confined ideal gas model. The level curves that form the phase portrait in Figure 1d correspond to different values of the integration constant $U_{0}$ and also of the values of the chemical potential μ. Sectors of these level curves that satisfy the boundary conditions in Equation (18) correspond to stationary states of our confined ideal gas. These sectors have a curvature $\pm \frac{1}{2}\left(\partial^{2}f/\partial \rho^{2}\right){|}_{\rho_{s}}$. Only some of these sectors have a negative curvature V(r)=−1/r. For example, one of them is the separatrix in Figure 1d.

**Figure 4:**
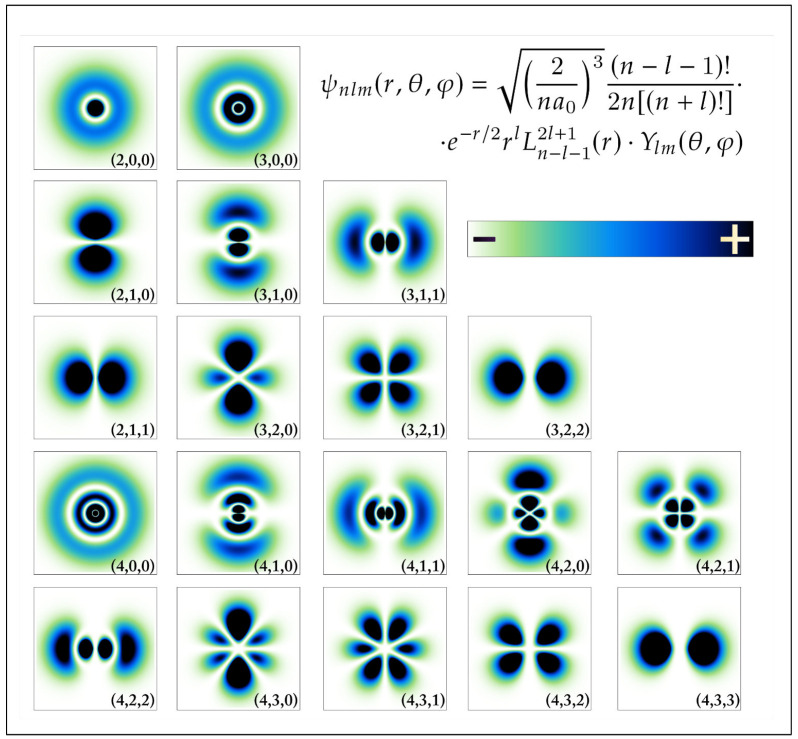
Two-dimensional graphical representations of the orbitals, wave functions $\psi_{n,l,m}\left(r,\theta ,\phi \right)$ of the hydrogen atom, as obtained from the single-electron time-independent Schrödinger equation. The functions L(r) and Y(θ,ϕ) are, respectively, Laguerre polynomials and spherical harmonics, *n*, *l* and *m* are quantum numbers. See text for details.

Clearly, the stability analysis of the confined ideal gas is provided by the Schrödinger Equation (8) with potential V(r)=−1/r. The resolution of this equation via separation of variables leads (besides factors we have ignored) to the well-known hydrogen atom eigenfunctions $\psi_{n,l,m}\left(r,\theta ,\phi \right))$ and their corresponding eigenvalues $E_{n,l,m}$. See Figure 4. As with the previous examples, the eigenfunctions $\psi_{n,l,m}\left(r,\theta ,\phi \right))$ represent specific kinds of ‘pure’ or ‘normal mode’ fluctuations of the confined ideal gas, and their meanings can be obtained through the analysis of their effect on the macroscopic stationary state $\rho_{s}\left(r;U_{0}\right)$. These normal-mode fluctuations can be given a specific order according to their progressive free energy cost given by the eigenvalue spectrum $E_{n,l,m}$. See Figure 4.

We have established a connection between the fluctuations of an ideal gas confined within a spherical cavity or shell (as described by a free energy density functional for nonuniform statistical-mechanical systems, Equation (1)) and the one-particle wave functions given by the time-independent Schrödinger equation for the hydrogen atom. But it is well-known that the set of eigenfunctions $\psi_{n,l,m}\left(r,\theta ,\phi \right))$ and their eigenvalues $E_{n,l,m}$ provide, beyond the one-particle formal scheme, the remarkable framework for the periodic table of atomic elements via the filling of eigenstates (with all understood modifications). Below, we point out briefly the extension path of our procedure that would involve more than one particle or, in this case, electrons. Of course, we ignore here molecular hydrogen, Pauli’s exclusion principle, etc. The statistical mechanics of the ideal gas is a limiting representation of a dilute noble gas (characterized by closed-shell electronic configurations).

Summing up, we have found explicit physical meaning for all the wave functions of the one-particle time-independent Schrödinger equation. Here we have only considered the wave functions for three of the well-known textbook problems, but this can be conducted for any one-particle potential function *V*(**r**).

## 5. Precisions on the Analogies Between Statistical Mechanics of Inhomogeneous Systems and One-Particle Mechanics

A prevailing working condition, say, when calculating one-particle mechanical properties, classical and quantum, is that the specific nature of the system is fixed, based on the selection, a particularization, of the potential function, both of the potential of force in Newton’s second law equation and the potential function in the time-independent Schrödinger’s equation. The same applies when the procedure is carried out via a classical Lagrangian or its equivalent version in quantum field theory. As we know, the transition from classical to quantum descriptions viewed through fixed potential functions is perplexing. But here, by following a statistical-mechanical description for inhomogeneous systems, we have encountered a different situation, the classical and quantum particle analogs do have a different nature, their potential functions are different, one being the curvature or second derivative of the other. Also, they become associated with different physical properties of the macroscopic inhomogeneous system.

The relationship between these two different potential functions in the mechanical analogies presented is a consequence of the particular statistical-mechanical approach we have chosen. This choice is of a phenomenological nature, the square-gradient free energy density functional for nonuniform systems. Remarkably, this functional leads (only) to single particle mechanical analogies, for which the basic mechanical laws are expressed by linear equations. As we have seen, the classical version corresponds to the macroscopic statistical-mechanical inhomogeneity, whereas the quantum case is linked to the fluctuations of that stationary state. The latter, as we know, is relevant, in general, only on microscopic scales. Going further, and reaching for a physical interpretation based on the exact analogies presented, we may venture that a quantum particle wave function is to be identified with a fluctuation of the inhomogeneous system. As we have detailed above, such fluctuation can be one of infinitely many possible variations given by a superposition of the eigenfunctions of the time-independent Schrödinger’s equation.

In order to identify the roles played by the two potential functions *U* and *V* we look back at the three examples presented in the previous Section. The classical particle trajectories obtained via the potential *U* merely describe the macroscopic stationary inhomogeneous states in the alternative language of classical mechanics, where the change in sign U(R)=−f(ρ)+μρ is the appropriate modification. On the other hand, the eigenfunctions and their associated eigenvalues obtained from the potential *V* point out the precise fluctuation normal modes responsible for macroscopic phenomena. Interestingly, the three single-particle quantum examples are best known for the following properties: (i) tunneling out of a potential well; (ii) zero-point energy and equally-spaced quantized energies; (iii) backbone for orbital and quantum numbers description of the periodic table of atomic elements. Are these properties related to the macroscopic inhomogeneous systems’ behavior?

In our chosen inhomogeneous system model examples, we have encountered and employed Helmholtz free energy density intervals with negative curvature in violation of the 2nd law of thermodynamics when the system is in a uniform state (a common feature when the mean-field approximation is adopted, as is the case with the square-gradient density functional we use). But, as pointed out, equilibrium nonuniform states often display this feature across their spatial structure and provide important measurable physical properties. In our discussion, the negative free energy curvature leads to an attractive particle potential *V* for the time-independent Schrödinger equation, a common feature of many quantum single-particle models.

Here we have followed a phenomenological approach, albeit a very successful procedure, to study statistical-mechanical inhomogeneous systems: the square-gradient density functional. The free energy density functional employed is based on van der Waals’ approach for spatially slowly-varying non-uniformities [32], which also, surprisingly, succeeds beyond such conditions. There are many statistical-mechanical studies of inhomogeneous systems that arrive at more realistic free-energy density functionals. There are even exact, first-principles derivations of classical density functionals for specific models [13,14,33]. These functionals are nonlinear and nonlocal. It is, therefore, worthwhile to examine the stability of the Euler–Lagrange solutions of these functionals to throw additional light on the analogies and their interpretations advanced here.

## 6. A Broader Discussion on the Physical Understandings of the Proposed Perspective, Additional Investigations Required, and Future Validations

Over the past century, many interpretations of quantum mechanics have been proposed. It is not our purpose here to provide a thorough examination of them, as their detailed descriptions and appraisals can be accessed elsewhere. We mention the names of the most relevant: Copenhagen interpretation, hidden-variable theories, many-worlds interpretation, Bohmian mechanics and pilot wave theory, quantum Bayesianism, quantum Darwinism, transactional interpretation, relational interpretation, etc. See Ref. [34] for brief summaries and pertinent web addresses. See also Ref. [35] for a recent survey with contemporary discussions and fitting references. These interpretations involve central notions concerning wavefunctions such as energy quantization, superposition, tunneling, and others. Interpretations have arisen under the belief that quantum mechanics is an incomplete theory, as some measurement puzzles, e.g., the wave function collapse [36], and the action at a distance or non-locality [37], have defied rationalizations. As we comment below, our framework delivers (in a speculative fashion) the above-quoted central notions from a new perspective and offers possible validations of the measurement problems. Even though we have limited the formal presentation to single-particle time-independent quantum systems, we can advance these assessments now.

### 6.1. Theoretical Constructions Built on Classical Statistics Concepts

Since our proposal is based on classical statistical mechanics, we compare ours with other schemes built on classical statistical concepts, for instance, the work of C. Wetterich [38,39]. We state the following distinctions: Our viewpoint rests strictly on the established statistical-mechanical procedure for inhomogeneous macroscopic systems and leads to the identification of quantum-mechanical wave functions as fluctuations inherent to the system’s canonical statistical-mechanical probability distributions. It differs from other studies in that the potential function that defines the nature of the quantum particles is not the same as that for the classical particles. It is also original because time evolution (of wave functions) is that of statistical-mechanical fluctuations (end of Section 3 and Section 6.4), not unitary time evolution as in the time-dependent Schrödinger equation. The new physical understanding of the wave functions of quantum mechanics (so far within the scope of the examples presented) is that each one of them plays a discernible, explicable role as a specific fluctuation of the macroscopic system.

### 6.2. Mathematical Analogy vs. Physical Interpretation

The distinction we offer between mathematical analogy and physical interpretation is that we indicate (so far, only for the single-particle time-independent case) a pathway to obtain physical understanding for each wave function. A simpler task when a general wave function is decomposed into normal mode wave functions (the opposite of superposition). The most visible physical interpretations are the roles played by the most prominent or singular wave functions (now specific fluctuation types) in macroscopic phenomena. We indicated/suggested that (i) tunneling of a particle in a box manifests as free interface displacement and phase change via nucleation. (ii) Harmonic oscillator zero-point energy assists profusion of critical fluctuations. (iii) Stability of hydrogen atom orbitals delivers scaffolding for noble gases. This, of course, requires further precise analysis. The following core issues of quantum mechanics are contained and rationalized by our statistical-mechanical viewpoint: (1) Superposition is a property fully shared by statistical-mechanical density fluctuations, as shown in terms of density fluctuation modes in Equations (7) and (11). (2) Measurement collapse can be naturally incorporated into the time evolution of statistical-mechanical fluctuations (wave functions) described at the end of Section 3. A measurement (here only considered as the initial condition perturbation of Equation (10)) would generally select some or even one fluctuation mode, then all modes collapse except the selected modes or a single mode. An example was given for nucleation in Section 4.1. Time evolution of density fluctuations described at the end Section 3 is of dissipative type. These are necessary, but feasible, continuations and directions of this line of work.

### 6.3. Extension to Many-Body Quantum Mechanics

The proposal can, on the whole, be extended to many-body wave functions. The density of an inhomogeneous system is the statistical-mechanical one-body probability distribution function, and we have identified density fluctuations as one-particle wave functions. Likewise, many-body wave functions would be identified with fluctuations of the many-body density probability distribution functions of the inhomogeneous macroscopic system. These distributions are well-defined and can be obtained. However, free-energy density functionals, like the square-gradient approximation, would require generalization. Entangled states occur in many-body quantum systems. Accordingly, and in relation to the above commentary, entanglement requires consideration of fluctuations of many-body density distribution functions of the inhomogeneous systems. We do not offer here viewpoints of entanglement phenomena from our framework, as this requires previous important developments. As a difference from the case of one-particle quantum problems, even time-independent many-body wave functions are not as readily obtainable from fluctuations of (stationary classical density functional) many-body probability distributions, as the density fluctuations of classical statistical-mechanical inhomogeneous systems.

### 6.4. From Stationary to Time-Dependent Quantum Phenomena

At the end of Section 3, we made use of the dissipative kinetic Equation (10), which originates from the works of Ginzburg and Landau [40], and we applied it to the square-gradient approximation to determine the density fluctuations’ time evolution. Consequently, and according to our framework, this choice makes wave function time evolution non-unitary. Alternatively, the density-conservative Cahn equation [40] can be used for the same purpose as we employed Equation (10) and turn the wave function time evolution unitary. Or, instead, one can explore the first-principles derivation of these two equations [40,41], and tune between the mentioned two limits with the use of more general kinetic equations [40,41]. Here we have only referred to real (number) wave functions, but the phase (of complex number) wave functions play an important role in quantum time-dependent phenomena. The counterpart of these properties in the dynamics of density (or many-body) fluctuations in classical statistical mechanics appears (to our knowledge) to be absent. Perhaps feedback from quantum-mechanical wave functions is useful here, and there is use of complex phases. If complex phases do not arise naturally from the proposed approach, this may indicate the need for additional structure in our framework for dynamical descriptions. Future work is needed to clarify these issues.

### 6.5. Beyond the Square-Gradient Approximation

The square-gradient approximation can be applied to many different inhomogeneous systems. And it was chosen here because it leads to the time-independent Schrödinger equation for a general potential function. There are many works on more accurate approximations or even exact free-energy density functionals, as in Refs. [13,14]. But the density functional expressions become more particular. The issue of generalized Schrödinger equations appears. This brings up the issue of nonlocality. Quantum mechanics allows nonlocal correlations. Actually, density fluctuations as obtained from some exact free energy functionals (as a difference from the square-gradient approximation) can display non-locality. See Ref. [13]. Another instance is given in Ref. [14], where phase change can result from correlated nonlocal nucleation sites. A closer look at this issue is needed as the connections, or differences, between non-locality in classical density functional instances and non-locality in quantum phenomena demand clarification before advancing our perspective in this and other directions.

Clearly, the above commentaries require additional investigations and future validations.

## 7. Closing Remarks

We have shown that the wave functions for the most common textbook problems in quantum mechanics—a particle in a well, the harmonic oscillator, and the hydrogen atom—are not purely abstract mathematical objects. Instead, they describe actual, physical fluctuations in macroscopic systems modeled via statistical mechanics: the distortions of a fluid interface, the critical fluctuations at a continuous phase transition or critical point, and the density modes in a confined ideal gas. The identification of the specific statistical-mechanical role for the solutions of the time-independent Schrödinger equation is a unifying perspective that has not received much attention. In plain words, we have delineated the following procedure: Consider the time-independent Schrödinger equation for any one-particle potential, solve it for its eigenvalues and eigenfunctions, and one can then systematically identify their role as descriptors of specific types of fluctuations of a precise inhomogeneous (in one coordinate direction) classical statistical-mechanical model system.

This perspective resolves naturally the dichotomy between macroscopic and microscopic descriptions: At large scales, probability distributions appear sharp (delta function-like), fluctuations do not play a significant role, and the world appears deterministic. The cancellation of the first variation in the free energy, the Euler–Lagrange equation, generates the observed macroscopic structures, which are elegantly translated into classical particle trajectories. At sufficiently small scales, where the observation of only a few particles is inevitable, the distributions appear broadened. Here, fluctuations dominate the physical picture, and their (static) features are precisely described by the (time-independent) Schrödinger equation for the microscopic potential. To reiterate, our main assertion in this study is that a one-particle wave function in quantum mechanics is a density fluctuation, or a fluctuation of the one-particle probability distribution function in an inhomogeneous system. And for multiple particles? The logical affirmation would be the following: The wave function is a fluctuation of the corresponding multiple-particle joint probability distribution function. Has quantum mechanics been running within statistical mechanics all the time?

## Data Availability

No new data were created or analyzed in this study. Data sharing is not applicable to this article.
